# Predictors of Disenrollment Among Medicare Fee-for-Service Beneficiaries With Dementia

**DOI:** 10.1093/geroni/igab046.062

**Published:** 2021-12-17

**Authors:** Maricruz Rivera-Hernandez, Aaron Castillo, Amal Trivedi

**Affiliations:** 1 Brown University School of Public Health, Providence, Rhode Island, United States; 2 Brown University, Providence, Rhode Island, United States

## Abstract

Medicare enrollment among people with Alzheimer’s Disease and Related Dementias (ADRD) has reached an all-time high with about 12% of beneficiaries having an ADRD diagnosis. The federal government has special interest in providing healthcare alternatives for Medicare beneficiaries. However, limited studies have focused on understanding disenrollment from fee-for-service, especially among those with high-needs. In this study we identified predictors of disenrollment among beneficiaries with ADRD. We used the 2017-2018 Medicare Master Beneficiary Summary File to determine enrollment, sociodemographic, clinical characteristics and healthcare utilization. We included all fee-for-service beneficiaries enrolled in 2017 who survived the first quarter of 2018. Our primary outcome was disenrollment from fee-for-service between 2017 and 2018. Regression models included age, sex, race/ethnicity, dually eligibility to Medicare and Medicaid, chronic and disabling conditions (categorized by quartiles), total health care costs including outpatient, inpatient, post-acute care and other costs (categorized by quartiles) and county fixed-effects. There were 1,797,047 beneficiaries enrolled in fee-for-service with an ADRD diagnosis. Stronger predictors of disenrollment included race/ethnicity and dual eligibility. Disenrollment rates were 7.9% (95% CI, 7.2 – 8.5) among African Americans, 6.6 (95% CI, 6.2 – 7.0) among Hispanics and 4.3 (95% CI, 4.2 – 4.3) among Whites. Duals were 1.9% (95% CI, 1.4 – 2.3) more likely to disenroll from fee-for-service to Medicare Advantage (MA). The inclusion of MA special need plans and additional benefits for those with ADRD and complex chronic conditions may be valuable for those beneficiaries with ADRD, and who may not have Medigap coverage when enrolling in fee-for-service.

